# Graph Partitions in Chemistry

**DOI:** 10.3390/e25111504

**Published:** 2023-10-31

**Authors:** Ioannis Michos, Vasilios Raptis

**Affiliations:** 1 Department of Computer Science and Engineering, School of Sciences, European University Cyprus, 6 Diogenous Str., Nicosia 2404, Cyprus; i.michos@euc.ac.cy; 2 Institute of Nanoscience and Nanotechnology, National Scientific Research Center ‘Demokritos’, Patr. Gregoriou E & 27 Neapoleos, 15341 Agia Paraskevi, Greece

**Keywords:** equitable partitions, externally equitable partitions, quotient graphs, molecular topology, information compression

## Abstract

We study partitions (equitable, externally equitable, or other) of graphs that describe physico-chemical systems at the atomic or molecular level; provide examples that show how these partitions are intimately related with symmetries of the systems; and discuss how such a link can further lead to insightful relations with the systems’ physical and chemical properties. We define a particular kind of graph partition, which we call Chemical Equitable Partition (CEP), accounting for chemical composition as well as connectivity and associate it with a quantitative measure of information reduction that accompanies its derivation. These concepts are applied to model molecular and crystalline solid systems, illustrating their potential as a means to classify atoms according to their chemical or crystallographic role. We also cluster materials in meaningful manners that take their microstructure into account and even correlate them with the materials’ physical properties.

## 1. Introduction

The concept of graph partition, stated in the context of algebraic graph theory, is applied to molecules and other chemical entities, and the implications are studied in the broader context of structure–property relations in materials. Graph partitions generalise the partitioning schemes based on groups of atoms defined by chemical reasoning (e.g., methyl, hydroxyl, carboxyl, etc.) and can be very insightful when it comes to the definition of groups of materials and the study of their properties. Some partitions can reveal symmetries and regularities that are not evident at first glance; others encode important chemical and topological information in a condensed manner. A special case of partitioning graphs that represent chemical entities is shown to combine several of the above features.

The building blocks of molecular, ionic and other materials are the atoms in their electrically neutral or charged state. We consider individual molecules as an example to introduce the core concepts of this work. Atoms in them are connected together through so-called covalent chemical bonds that can be of single or multiple order (usually up to three). This is certainly reminiscent of a weighted graph where atoms are the nodes and bonds play the role of edges with bond order being the edge weight. Thus, graphs come up as an entirely natural description of chemical entities. In fact, this representation is well-known to chemists who call it *syntactic formula* of a given molecule. Depending on its kind (the chemical element it belongs to) an atom can be bonded to a specific number of other atoms (thus hydrogen is linked to just one other atom, carbon can be linked to up to four atoms, and so on). This is the valence of the chemical element and determines the graph-theoretical valence or node degree. Sometimes, atoms of the same chemical element can have different valence, depending on the atom’s ‘chemical environment’. From a graph-theoretical perspective, these instances of the same element can be distinguished and taken as different kinds of atoms.

Atoms can be arranged in an orderly manner in space, especially in infinite periodic crystal solids where a basic unit containing a finite number of them, the *unit cell*, is repeated in all directions, tiling the whole space. This gives rise to infinite periodic graphs that can be represented by the graph of their unit cell subjected to toroidal boundary conditions, as explained in subsequent paragraphs. In these materials, atoms are arranged in space in very specific ways thanks to the balance of total repulsive and attractive forces among them. Quite often, these atoms are not bonded through covalent bonds as in molecules. However, from a graph-theoretical viewpoint, we can consider the nearest neighbours of each atom to be ‘bonded’ to it, so a graph can be constructed to model the material. The number of these nearest neighbours, known in chemistry as *coordination number*, is the node’s degree.

We often find that atoms tend to gather in specific groups that are repeated many times and combined with each other to form the material. Some of these groups play an important role in chemistry and physical chemistry; they are called *functional groups*. Others are building blocks that can be repeated and recombined in many ways to form different kinds of all molecules. By way of examples, linear hydrocarbons such as butane or octane consist of groups called *methyl* (one carbon and three hydrogens) and *methylene* (one carbon, two hydrogens); ethanol contains a methyl, a methylene and the functional group of *hydroxyl* (just one oxygen and one hydrogen atom) and so on.

Then, it is customary, when looking at a molecule and studying its properties, to consider it as an assemblage of such groups. We can still use a graph (or syntactic formula, in the chemists’ language) to draw a molecule using its groups rather than atoms as building blocks. Chemically, these are ‘coarse-grain’ graphs that neglect some details to the extent to which they do not matter much. Mathematically, this ‘group decomposition’ of a molecule is a partition that is well known to chemists and makes sense to them because these groups are intimately related with the physical and chemical properties of the materials. However, as we intend to show, many other partition schemes are also possible and could give rise to insightful relations connecting the structure and topology of materials with their properties.

The concept of *quotient graph* in a broad sense can be introduced here by considering a partition scheme *P*, which gives rise to a coarser representation of a molecule, e.g., when using groups of connected atoms instead of the atoms themselves. The particular ways in which the nodes of the quotient graph are connected are specified subsequently, with regards to the nature of the system and the partition applied to it.

Chemists are tempted to partition molecules in terms of functional groups and to define quotient graphs accordingly. This approach has been fruitfully exploited by Hajiabolhassan et al. [1] to improve application of graph neural networks in the prediction of molecular properties, being able to condense the original molecular graphs to much smaller sizes while retaining essential information about their chemistry.

In the literature concerning solid materials and crystal structures, the term ‘quotient graph’ has been used to describe specifically the contraction operations that map an infinite periodic structure to a finite graph that preserves all essential information about the structure and connectivity of the crystal’s fundamental periodic units. The relevant definitions take advantage of the system’s translational symmetry to do so. One such definition is employed in a line of research that was initiated by Chung et al. [2], who termed their approach the ‘vector method’. According to it, an infinite three-dimensional periodic lattice is partitioned into sublattices, each of them containing all periodic replicas of a lattice point of the minimum unit that can reproduce the whole lattice by repeating itself via translation symmetries. The sublattices are mapped to corresponding nodes of the quotient graph, while the edges in it are defined by all possible connections between any inter- or intra-sublattice point pairs, respecting the lattice structure and periodicity. A multigraph with loops can arise as a result of this process in the general case. This quotient graph is completed by annotating the edges with triplets of integers to indicate whether the node pairs represent lattice point pairs within a translational periodic unit or ones shared by neighbouring units along all three translation directions. This approach is useful, among others, in devising algorithms for the computational prediction and design of crystal structures, as Gao et al. have shown [3].

Another similar way to define finite graphs for periodic crystal materials is based on the partition of space into what is known as the crystal *unit cell*, i.e., a volume element containing an adequate subset of lattice points, which can tile the space and reproduce the whole lattice. In this case, the so-called *toroidal* or *periodic boundary conditions* allow one to define edges between atoms that are situated far apart within the unit cell but linked to periodic replicas of each other residing in the adjacent unit cell copies (a simple example is given in the next section). The validity of toroidal conditions as an adequate description of periodic systems has a sound basis in solid-state physics [4], and their usage is also extensive by the material modelling community [5]. The so-called minimum image convention [5] can then be used to choose the copy closest to a given atom among all the periodic replicas of another nearby site and define their pair in a unique unambiguous manner. This technique can then be used to define edges of a graph where its nodes correspond to the atoms in the unit cell (a recent work claims to use this approach to define unit cells, in a broader sense, even for nonperiodic quasicrystals [6]).

Apart from the above approaches, there are many more ways to partition a system, such as infinite periodic, finite molecular or others. From a purely algebraic standpoint, a work by Neuberger et al. [7] summarises neatly the underlying framework in terms of invariant subspaces of suitably selected matrices that can be readily transferred to the adjacency, Laplacian and various other matrices that describe graphs.

In this work, we use an alternative scheme for the contraction of a finite graph that describes a material system at the molecular and atomic level. This scheme emanates from the concept of *equitable partition* (EP), which is intimately related with the system’s underlying generalised symmetries. (The first author of the present work has successfully used in the past such a notion to condense the directed graph of cliques of a trace monoid that represented a parallel system [8]). Importantly, two or more kinds of partitions and corresponding methods for the extraction of quotient graphs can be combined together, e.g., functional group-based together with equitable partitioning. In a similar vein, the above-described quotient graphs of periodic systems are, in fact, used by us as a starting point for further partitioning and contraction of the graph that describes a given material.

In the following sections, we consider finite graphs *G* that describe either the connectivity of atoms in a molecule or the coordination of atoms in unit cells subjected to toroidal conditions, as described above; all of them are collectively termed ‘molecular graphs’, for the sake of simplicity.

We consider a simple (undirected, loopless, lacking multiple edges) graph G(V,E), where *V* is the set of vertices and *E* is the set of edges. An *externally equitable partition* (EEP) $P=\{C_{1},\ldots ,C_{i},\ldots ,C_{j},\ldots ,C_{p}\}$ is a partition of *V* with the property that every vertex of each ‘cell’ $C_{i}\subseteq V$ is connected with the same fixed number $b_{ij}$ of vertices in another cell $C_{j}$, for all i≠j [9]. An EEP is *equitable* (EP) when the same condition holds among elements of the same cell. Given an EEP *P* of a graph *G*, the *quotient graph* QG(P) is the weighted directed graph, with the cells of *P* as its nodes and with an arc of weight $b_{ij}$ from cell $C_{i}$ to cell $C_{j}$ for all i≠j. When *P* is an EP, arcs in the form of loops are also possible for i=j. EP-derived quotient graphs are akin to the so-called divisors of graphs in [10]. In this work, we also introduce a special case of (E)EP, termed Chemical Equitable Partition. Its definition is given in detail in the next section.

## 2. Materials and Methods

### 2.1. Molecular Systems

In this subsection, we consider individual molecules without boundary conditions and we introduce a specific case of an EEP (which turns out to be an EP) that is referred to as *Chemical Equitable Partition* (CEP). This is defined by starting from the initial partition of the atoms in the molecule, based on their valence and chemical element, and then iteratively applying steps similar to those in an algorithm described in [11], leading to a specific partition which, in the general case, further divides the atoms of identical chemical elements into subgroups of nodes sharing common connectivity profiles.

We give a formal definition of CEP before presenting a simple algorithm to derive Chemical Equitable Partitions of molecular systems.

**Definition** **1** (Chemical Equitable Partition)**.** *Let there be a graph G(V,E), with the partition D of its vertices grouped by their degree, and a refinement of D, referred to as ‘Chemical Partition’, CP. A partition P of G is termed* Chemical Equitable Partition *if the following are true:*

- *It is the coarsest, not necessarily unique, EEP of G;*
- *Additionally, it is a refinement of CP.*

The term ‘Chemical Partition’ obviously originates from the wider context of the problems discussed in the present article. Thus, the CP of a molecular graph can be one in which atoms are partitioned according to their valence and chemical element. Similar chemical partitions can also be defined when considering groups of neighbouring atoms, as the basic building blocks of a molecule.

**Figure 1:**
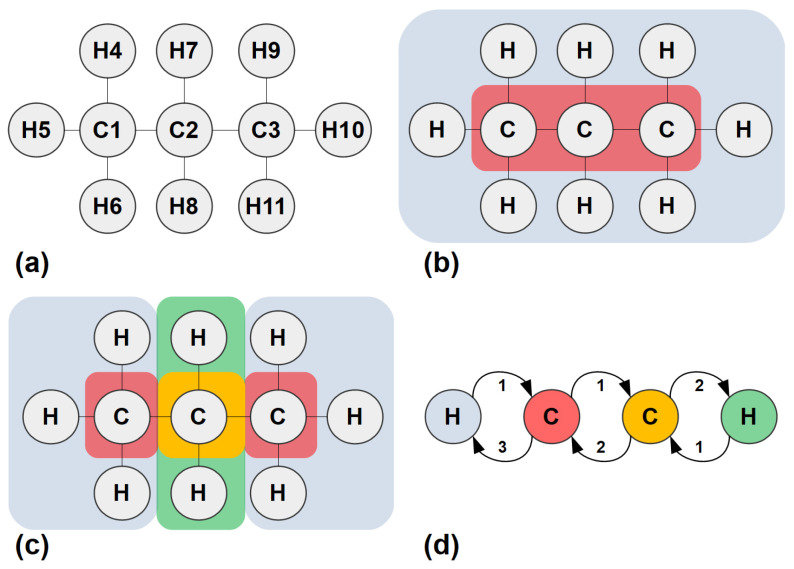
Definition of CEP and an algorithm for its derivation. (**a**) Example: n-propane. (**b**) Atoms split into cells, according to their chemical element and node degree (in the case of molecular graphs: chemical valence). (**c**) Split further, as in O’Clery (2013) [11], until eliminating all violations of same cell-to-cell out-degree condition. The resulting EEP is easily shown to be an EP. (**d**) Quotient graph, QG(CEP): Nodes are classified according to their connectivity to their surroundings.

A simple step-by-step example serves to illustrate the concept of CEP and its application to molecular and ionic systems. We consider n-propane, a simple hydrocarbon molecule, with its syntactic formula shown in Figure 1a. It consists of a linear backbone chain of three carbon atoms (C), numbered 1 to 3, connected to eight hydrogen atoms (H), numbered 4 to 11. In graph-theoretical terms, carbon and hydrogen atoms are nodes of degrees 4 and 1, respectively, equal to their chemical valence; these are adjacent when the atoms are connected by single covalent bonds, which form the graph edges with weight equal to 1. The molecule resembles a tree graph, but the concepts and steps illustrated below are applicable to physico-chemical systems of arbitrary topology.

An algorithm for determining the CEP of a given molecule consists of three kinds of steps. First, the partition is initialised. Then, steps, alternating in pairs, follow, determining ‘connectivity profiles’ for the nodes and re-defining the molecular partition based on these profiles. Finally, the concept of CEP is applied to n-propane as follows (see also Table 1):

**Step 0** (Initialisation): The graph nodes or atoms are partitioned according to the corresponding valence and chemical element. Thus, we come up with two partitions or ‘cells’, one of them containing all carbon atoms, and one containing all hydrogen atoms, as shown in Figure 1b. The cells are numbered 1 and 2 and the corresponding column, ‘Step 0’, in Table 1, is filled accordingly;

**Step 1** (Node profiles): Following a process similar to the one described by O’Clery [11], we add two columns next to the Step 0 column, corresponding to the two cells, under the common header ‘Step 1’. Their entries are filled as follows: for each atom or node *i* from 1 to 11, the number of edges going to nodes belonging to each cell *j*, from 1 to 2, is introduced in the corresponding column’s entry. Thus, carbon number 1 is connected to one node in cell 1 (the carbon atom number 2, adjacent to it) and three nodes in cell 2 (hydrogen atoms 4 to 6). The rest of the entries are filled in a similar manner;

**Step 2** (Re-partitioning): Nodes are regrouped according to their ‘connectivity profile’ formed by the cell columns, i.e., all nodes sharing identical rows under the previous step’s header, are said to belong to the same cell, and cells are redefined accordingly. In this example, the end carbons form cell 1, the middle carbon belongs to cell 2, and all hydrogen atoms comprise cell 3. If the new partition coincides with the last one, the algorithm is terminated; else, the last two steps are repeated;

**Step 3** (Node profiles): In this example, the new partition differs, so we re-iterate the procedure of finding node connectivity profiles. Three cells have been defined; therefore, three columns are added and the corresponding profiles are determined;

**Step 4** (Re-partitioning): New cells are defined according to the most recent profiles. This time, hydrogen atoms are split into two cells according to whether they are bonded to the middle or one of the end carbons.

**Step 5** (Node profiles): The partition determined in the last step differs from the one in Step 2 and features four cells instead of three, so new node profiles have to be determined;

**Step 6** (Re-partitioning): The new molecular partition defined on the basis of the last node profiles, Step 5, is identical to the one in Step 4. Therefore, the algorithm is terminated.

The final cells are as shown in Figure 1c. The corresponding quotient graph is the weighted directed graph of Figure 1d, depicting the connectivity of the cells. The above-determined partition is an EEP by construction, with the additional merit that it takes the chemical composition into account since it started by splitting the atoms according to their chemical elements.

An important point has to be mentioned here: unlike existing versions of chemical graph theory [12], the degree of chemical bonds is not ignored. Thus, for instance, double and triple carbon–carbon bonds correspond to graph edges with weight 2 and 3, respectively, whereas the aromatic carbon–carbon bonds in such a molecule as benzene are mapped to edges with a fractional weight of 1.5; otherwise, important chemical information can be lost. It is also worth noticing that the same procedure can be carried out using functional groups or coarse-grain ‘united atoms’ instead of atoms, e.g., methyl and methylene groups.

Finally, it is noted that, as in [11], the above-described algorithm generates the coarsest possible EEP given the initial partition, according to valences and chemical elements. Now, we proceed to show that an EEP defined via the above algorithm, i.e., a CEP, is also an EP.

**Proposition** **1.** *The CEP of an individual molecule is an EP.*

**Proof.**  Let us consider a cell $C_{i}$ of the CEP of a molecule partitioned into *p* cells, as in the above-described procedure. For a given vertex *v* of *G* in $C_{i}$, its degree equals

$$d_{v}=\sum_{\begin{array}{c} j=1\\ j\neq i \end{array}}^{p}d_{vj}+d_{vi},$$

where $d_{vj}$ denotes the number of edges going from *v* to elements of other cells $C_{j}$ (inter-cell edges), according to the given partition, and $d_{vi}$ is the number of remaining edges going to elements *w* in $C_{i}$ itself (intra-cell edges). By construction, our CEP is one in which all vertices in a given cell *C* have the same degree $d_{C}$ (the valence of their chemical element); therefore, for every *v* in $C_{i}$, we obtain

$$d_{vi}=d_{C_{i}}-\sum_{\begin{array}{c} j=1\\ j\neq i \end{array}}^{p}d_{vj},$$

so that the intra-$C_{i}$ subgraph is $d_{vi}$-regular. □

### 2.2. Crystalline Solids and Toroidal Conditions

The above example showed how to extract the CEP of individual molecules with a well-defined structure, based on covalent chemical bonding of atoms. Another example serves to extend the applicability of the above scheme to different kinds of materials like crystalline solids. In this case, the material consists of a so-called unit cell (of cubic, parallelepiped or other form, characterised by three main axes) which contains a number of atoms of various chemical elements in well-defined fully occupied positions (situations such as partial and mixed occupancy are routinely encountered by crystallographers, but they would require a treatment of statistical nature and are not considered in this work). The material is built by replicating the unit cell along its axes so as to tile the entire space.

Atoms in such materials as ionic crystals or metals are usually held together by means of non-directional electrostatic forces among ions or ions and free electrons, instead of covalent bonds that form molecules. There are also cases of crystals formed by means of covalent bonds as for instance carbon allotropes (graphite, diamond) or silicon carbide, or even other kinds of interactions (for an overview of the kinds of crystals with respect to the forces that form them see [13]). Still, the result is the same in the sense of atoms ordered in a regular manner in space, which can be described by the replication of a basic unit along all directions. In all these periodic systems, adjacency can be defined in another manner. For each chemical element in the system, we consider a radius of the corresponding atoms (assuming they are of spherical shape). Then, we use these radii to detect overlapping atom pairs that are marked as adjacent. Non-overlapping atom pairs are considered non-adjacent. The radii are defined such that, for each atom, we determine its nearest neighbours. The number of nearest neighbours of a given atom is known as its *coordination number*.

**Figure 2:**
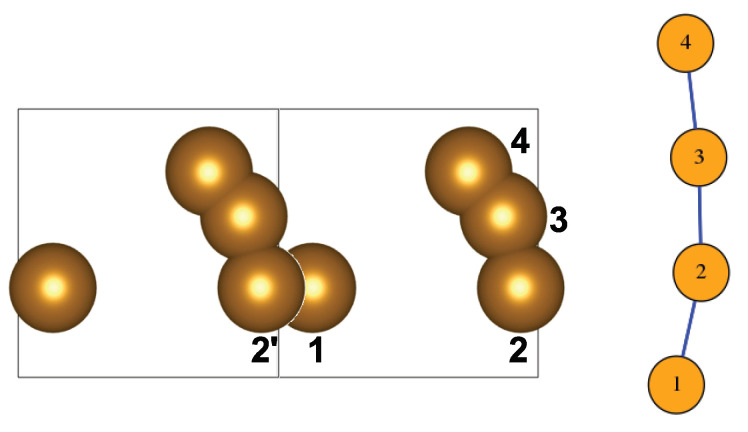
A fictitious unit cell with four atoms, illustrating the definition of adjacency under toroidal conditions. Atom pairs 2–3 and 3–4 overlap; thus, they are adjacent. Atoms 1 and 2 do not overlap; however, atom 1 overlaps with the periodic image 2′ of 2 on the left. Thus, atoms 1 and 2 are adjacent too. This is illustrated in the resultant path graph on the right.

When calculating atom pair distances to compare them with the sum of the atoms’ radii, the cell’s periodicity has to be taken into account. Thus, a given atom near a face of the cell will also have neighbours from the adjacent replica of the cell. These neighbours are ‘periodic images’ of atoms of the unit cell that may appear to be situated far apart within that cell (i.e., near the opposite face). An example is shown in Figure 2. The cell contains four atoms numbered 1 to 4, of which atoms 2,3,4, near the rightmost face, form two overlapping pairs (2–3 and 3–4), while atom 1, near the leftmost face, does not overlap with any of them. However, the replica of the unit cell, on the left, contains a copy of atom 2 (denoted as 2′) which overlaps with 1. Then, we also consider atoms 1 and 2 as adjacent and obtain the graph shown in the figure. This way of defining adjacency, i.e., selecting the nearest neighbour $b_{\mathrm{prox}}$ to an atom *a* among all the periodic images of an atom *b*, constitutes the so-called *minimum image convention* and is an essential part of the way *periodic boundary conditions* are commonly employed in many computational applications [5].

**Figure 3:**
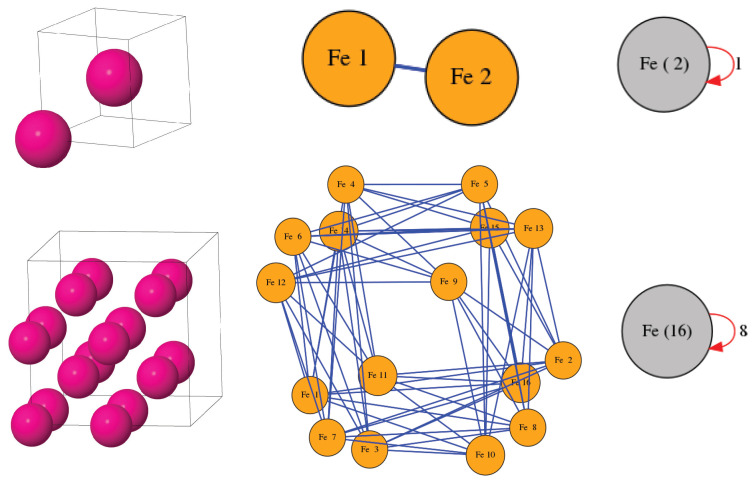
Body-centered cubic iron (bcc Fe) as an example of a crystalline periodic material. **Top** (left to right): bcc iron unit cell; graph of the cell under toroidal conditions; corresponding quotient graph. **Bottom** (left to right): Same for a 2×2×2 supercell replicating the unit cell twice along each axis. Note: Numbers in parentheses in the quotient graph cells denote the numbers of graph nodes grouped together in the corresponding cells.

As a concrete example of crystalline materials, we look at the micro-structure of iron. Under ambient conditions, iron crystallises into a body-centered cubic structure with lattice constant a=0.2861nm, as shown in Figure 3. The unit cell is a cube that contains two atoms, one of them occupying a corner of the cell and the other one situated in the centre. By replicating this cell along its axes, the whole space is tiled. Also, the other seven corners of each cell are occupied by the atoms of the neighbouring cells. It is easy to see that each atom in the lattice has exactly eight nearest neighbours and, thus, a coordination number equal to eight. Given the appropriate toroidal conditions, we come up with a simple graph, which, in this case, turns out to be a path graph with two nodes corresponding to the two atoms. Applying the procedure outlined in the case of propane, it is very easy to come up with the quotient graph, also shown in Figure 3. This quotient graph contains just one cell encompassing all the atoms in the system and corresponds to a so-called *trivial equitable partition*. Since all nodes are adjacent to nodes belonging in that same cell, there is just one arc in the form of a loop, with weight equal to 1.

The above construction is mathematically sound but suffers from one drawback: the node degree in the original graph and the arc degree in the quotient graph do not agree with the actual coordination number of the atoms. Thus, some important physical information has been lost in the process. To address this issue, we now consider a so-called supercell obtained by replicating the unit cell a certain number of times along the cell axes. Here, we consider the 2×2×2 supercell in which all atoms are surrounded by eight nearest neighbours, as in the actual material. Indeed, all nodes of the resultant graph shown in the bottom row of Figure 3 have the same degree (8) coinciding with bcc iron’s coordination number—this is also the weight of the arc in the corresponding quotient graph, which, once again, corresponds to a trivial equitable partition.

The above discussion shows that the procedure of extracting the CEP of a given chemical system can be easily extended to crystalline systems, but care should be taken when selecting the appropriate unit cell to determine the system’s graph and quotient graph. A distinction exists in crystallography between the conventional unit cell and the primitive cell of a system. The latter is the smallest possible cell that reproduces the lattice when appropriately replicated to cover the whole space. However, the full symmetry of the system is not always apparent through the primitive cell and the conventional unit cell is commonly used to reveal that symmetry. Conventional unit cells may contain more than one primitive cells and can be large enough to replicate the actual local environment of each atom so that node degrees and arc weights are consistent with the coordination numbers and chemical valences. Thus, they are a safe choice in many cases when trying to extract graphs and quotient graphs capable of encoding physically meaningful information.

Concisely, the procedure to extract the CEP of an infinite periodic crystal includes the following:

1. Imposing periodic boundary conditions, with the aid of the minimum image convention, to the unit cells under consideration;
2. Taking the conventional unit cell or defining a large enough supercell, as discussed above;
3. Deriving the finite graph *G* of the chosen cell by detecting nearest neighbours of atoms under the above boundary conditions.

**Proposition** **2.** *The CEP of an infinite periodic crystal derived under the above conditions (1 to 3) is an EP.*

**Proof.**  The minimum image convention implies that any vertex is adjacent to a well-defined number of nearest neighbours, equal to the coordination number dictated by the nature of the corresponding atom (chemical element, crystallographic position, immediate ‘chemical environment’). Then, the same logic as in the case of individual molecules applies to the finite graph *G* of the unit cell under consideration, and its CEP, under the above conditions, is an EP. □

### 2.3. Equitable Partitions as Information Compression Operations

The usage of different kinds of cells when partitioning the microstructure of crystalline materials brings to the fore another aspect with interesting implications. A conventional unit cell or a supercell may contain redundant information about the lattice structure as compared to the primitive cell. Other kinds of information redundancy are associated with the symmetries or regularities present in the system. These symmetries correspond to the automorphism group of the corresponding graph. When determining the CEP (or the (E)EP in general) an information reduction operation is actually carried out.

Let *P* be a CEP or another (E)EP partition of a molecular or crystalline material graph, *G*. The simpler the QG(P), the richer the symmetry of the original graph; thus, (E)EP, and its associated QG, pave the route to a characterisation of a graph’s symmetry. Some of the information related to the original graph is lost in the partitioning process, but an important part thereof (a subset of the spectrum) is transferred invariably to the QG [9,10]. Thus, an (externally) equitable partition can be thought of as a lossy compression scheme. In this respect, it would be interesting to quantify the information compression associated with CEP, or (E)EP in general, as a means to quantify the symmetries of the graph that describes a system.

An attempt to quantify redundancy in graphs with respect to their equitable partitions has already been described by [14]. Alternative definitions were given, therein, for the cases of sparse graphs and strongly nonsparse graphs, with the information compression expected to vary between these two extremes in the general case. In the present work, we quantify the information compression associated with an (E)EP in the general case by defining a *compression ratio* based on the building blocks of the original and quotient graph, namely, their nodes and edges or arcs. A crude compression measure would be simply to divide the number of nodes in the QG by the number of nodes in G (much akin to the ‘abstraction ratio’ defined by Hajiabolhassan et al. in [1]). The arcs of the QG, however, introduce an extra layer of complexity that offsets, to some extent, the information compression quantified by the nodes. We take this into account by introducing the ratio of the arcs over edges alongside the corresponding % ratio of the nodes in our definition.

Then, we come up with the following expression for the compression ratio in the general case (regardless of the graph sparsity):

(1) $$\%\;CR=100\cdot \frac{n_{QG}}{n_{G}}\cdot \frac{a_{QG}}{m_{G}},$$

where $n_{G}$ and $n_{QG}$ are the numbers of nodes; $m_{G}$ is the number of edges in the original (undirected) graph *G* and $a_{QG}$ is the number of arcs in the (generally directed) quotient graph QG(P). The ratio $100\cdot n_{QG}/n_{G}$ expresses the % decrease in graph size; $a_{QG}/m_{G}$ accounts for the increase in complexity associated with the edges and arcs of the graph and the quotient graph, respectively.

Finally, to account for the information redundancy arising when using larger than primitive cells for crystalline materials, a factor is introduced to counterbalance this kind of information excess. Thus, the fully fledged definition used in our calculations is given by the following expression [15]:

(2) $$\%\;CR=100\cdot \frac{n_{QG}}{n_{G}}\cdot \frac{a_{QG}}{m_{G}}\cdot Z\cdot f.$$

The Z·f product concerns crystalline materials that exhibit periodicity, and their finite contracted graph has been derived from the material’s conventional unit cell; otherwise, it is set to 1. Thus, *Z* is the number of formula units in the conventional unit cell [16] and *f* is the number of lattice points per cell [17]. In other words, Z·f is a reweighting factor that renders the compression ratio consistent with the smallest unit that, under the appropriate symmetry operations, can reconstruct the system (reduced cell) when the conventional unit cell has been used to derive the structural graph. Molecules in isolation and amorphous cells subjected to periodic boundary conditions, like the ones used in molecular simulations, do not need this kind of reweighting (Z·f=1).

## 3. Results

### 3.1. Examples of Molecular Systems

**Figure 4:**
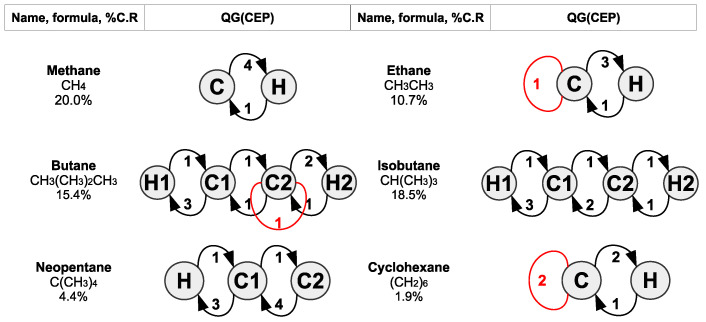
Examples of molecular and quotient graphs of saturated linear, branched and cyclic hydrocarbons. Subscripts in the condensed syntactic formulae denote the number of atoms of a given type, whereas non-subscripted numbers in QG(CEP) graphs distinguish among different cells consisting of atoms of the same chemical element. Loops that turn CEPs from EEPs to EPs are coloured red. Compression ratios have been calculated according to Equation (1).

Now, we are ready to apply the procedures described in the previous section to look at the chemical equitable partitions of molecular and crystal systems and gain insights about their microstructure. First, we look at the prototypical example of *saturated hydrocarbons*, which are simple in terms of chemical composition and yet can have a rich topology (though the aforementioned concepts are readily applicable to chemical systems of arbitrary composition and structure). Figure 4 shows the syntactic formulae and CEP-quotient graphs of some linear, branched and cyclic hydrocarbons. The corresponding compression ratios obtained from Equation (1) are also shown.

Methane has the highest compression ratio as it is the smallest and simplest of all hydrocarbons, and, as such, it does not leave room for substantial compression as compared to other molecules of the same family. As we move to larger and more complicated molecules, though, we observe that the highly symmetrical neopentane, with five carbon atoms, exhibits a remarkably low CR of 4.4%, as compared to 15.4% and 18.5% of smaller (four carbon atoms) but less symmetrical butane and isobutane, respectively. If we write their syntactic formulae in a planar form, the latter two would have a $C_{2}$ symmetry, whereas neopentane features a $C_{4}$ symmetry. The more that atoms are unchanged under the symmetry operations characterising a molecule, the more condensed its QG(CEP) will be.

Remarkably, there is a one-to-one correspondence between linear or branched saturated hydrocarbons and their QG(CEP) graphs, as can be shown easily. On the contrary, cyclohexane is *not* unambiguously determined by its QG(CEP); the same quotient graph can be obtained using any other saturated cyclic hydrocarbon. Although such molecules have a $C_{n}$ symmetry, where *n* is the number of carbons, their closed-loop form allows us to look at them as linear chains subjected to periodic boundary conditions. Here, methylene, -CH${}_{2}$-, is the basic periodic unit bonded to two adjacent identical groups of atoms. In this way, cyclic saturated hydrocarbons can be said to exhibit translational symmetry along their carbon–carbon chain, which is broken in the case of (finite) linear or branched hydrocarbons. Of note, the same quotient graph as QG(CEP) of cyclohexane can be obtained by applying CEP to ethylene, H${}_{2}$C=CH${}_{2}$, if we stick to our convention that double chemical bonds correspond to molecular graph edges with a weight of 2.

### 3.2. CEP as a Classifier of Atoms

The effect of broken translational symmetry is to further differentiate graph nodes with respect to their placement relative to other existing symmetry elements like inversion centres or mirror planes. This can be seen in a simple yet instructive example: the generic case of linear saturated hydrocarbons or n-alkanes. These are simple chain molecules consisting of methylene, -CH${}_{2}$-, groups bonded together in a row and two methyl, -CH${}_{3}$-, groups situated at the two ends of the chain. To further simplify the problem, we take advantage of the previously mentioned fact that (E)EPs can be combined with other partitioning schemes by, for instance, breaking down into functional groups and other subsets of atoms connected together. In this example, we adopt a ‘united-atom’ approach by viewing all methyl and methylene groups as single structureless sites. In this simplified representation, a given alkane chain containing *n* carbon atoms will consist of n−2 methylene sites and 2 methyl sites situated at its two ends, instead of *n* carbon atoms and 2n+2 hydrogen atoms in the ‘fully atomistic’ case.

**Figure 5:**
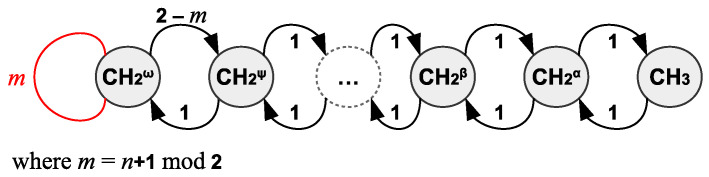
General form of QG(CEP) of linear saturated hydrocarbons with more than two carbons. Greek letters denote cells with chemically identical groups that differ by place and connectivity.

Figure 5 displays the CEP of linear alkanes in general in their united atom representation. Each cell contains exactly two groups, except the one marked with an ω, which contains one or two groups, depending on *n*, the number of carbons, being odd or even, respectively. Overall, there are $m=\lceil \frac{n}{2}\rceil$ cells in QG(CEP). For odd *n*, we obtain a loopless path graph, with arcs of weight 1, except one that has weight 2, whereas, for even *n*, QG(CEP) has a loop at the end and all its arcs have weight 1. The derivation and significance of this quotient graph can be explained by the following reasoning.

A linear alkane containing *n* carbon atoms can be written as

$${\mathrm{CH}}_{3}-{\mathrm{CH}}_{2}^{\alpha }-{\mathrm{CH}}_{2}^{\beta }-\cdots -{\mathrm{CH}}_{2}^{\psi }-{\mathrm{CH}}_{2}^{\omega }-{\mathrm{CH}}_{2}^{\omega }-{\mathrm{CH}}_{2}^{\psi }-\cdots -{\mathrm{CH}}_{2}^{\beta }-{\mathrm{CH}}_{2}^{\alpha }-{\mathrm{CH}}_{3}$$

if *n* is even, or

$${\mathrm{CH}}_{3}-{\mathrm{CH}}_{2}^{\alpha }-{\mathrm{CH}}_{2}^{\beta }-\cdots -{\mathrm{CH}}_{2}^{\psi }-{\mathrm{CH}}_{2}^{\omega }-{\mathrm{CH}}_{2}^{\psi }-\cdots -{\mathrm{CH}}_{2}^{\beta }-{\mathrm{CH}}_{2}^{\alpha }-{\mathrm{CH}}_{3}$$

if *n* is odd, where superscripts α, β, ⋯, ω denote positions of methylene groups relative to the methyl ones. The difference among groups α, β, ⋯ and so on becomes evident when we consider the possibility of a substitution reaction in which a hydrogen atom of a methylene group is replaced with a chlorine (or other haloge)n atom. Thus, for instance, said substitution in α or β methylene groups of n-pentane will yield 2-chloro-pentane and 3-chloro-pentane, respectively. This is in stark contrast with cycloalkanes, where all methylene groups are equivalent and substitution of one hydrogen atom anywhere on the ring molecule will yield the same product due to the high symmetry of the reactant. CEP will group all chemically equivalent atoms together in their respective partition cells, as shown in Figure 5 (also, compare with cyclohexane in Figure 4 as a case where all carbon atoms are equivalent); thus, CEP is a *classification* scheme. Furthermore, this scheme works for arbitrary system topologies, thus opening a new way to extract ‘chemically meaningful’ information from systems of any size and complexity.

### 3.3. Examples of Solid Crystals

**Figure 6:**
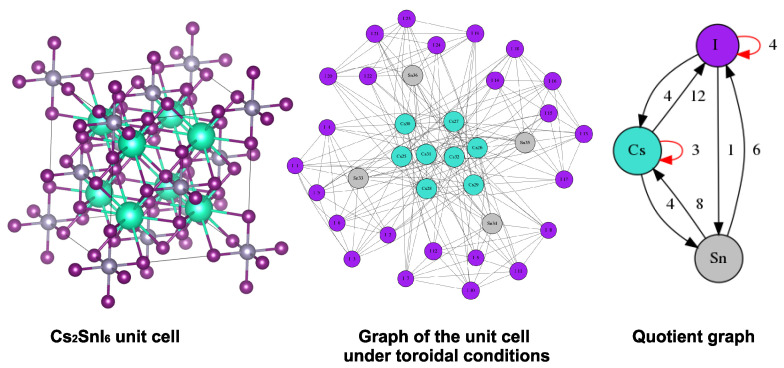
Unit cell and quotient graph of an inorganic perovskite, Cs${}_{2}$SnI${}_{6}$. Number of formula units in conventional cell, Z = 4; lattice points per cell, f = 4; compression ratio: 5.03%.

The above examples concerned molecular systems with well-defined chemical bonds connecting the atoms. The same concepts hold, though, for solid-state crystal systems, where the adjacency is defined with the aid of overlapping atom pairs of nearest neighbours. Figure 6 shows the conventional unit cell of a so-called defect perovskite, Cs${}_{2}$SnI${}_{6}$ [18], the corresponding graph and the resultant QG(CEP) graph. The material has a rich symmetry, which is also reflected in its finite contracted graph, as evidenced by the relatively small and simple quotient graph, and a compression ratio of 5.03%. Adjacent atom pairs have been identified by determining overlaps when assigning the material’s chemical elements with the van der Waals radii proposed by Alvarez [19]. The atoms remain partitioned according to their chemical element, and no further splitting to same-element subsets took place.

An interesting class of solid materials includes crystals consisting of an inorganic framework enclosing or combined with organic ions or molecules, such as hybrid organic–inorganic perovskites and metal–organic frameworks (MOF). The coexistence of a molecule-like part having well-defined chemical bonds and an inorganic part where atoms are coordinated together via non-directional forces calls for a careful definition of the adjacency criterion. However, mixing the two kinds of species in a periodic lattice does not pose any serious difficulty. We simply observe that bonded atom pairs always overlap if we use large enough atom radii, appropriate for the detection of non-bonded overlapping atom pairs. In fact, bonded atom pairs are generally closer than non-bonded ones—even more so for bonds of higher than single order. Thus, we are entitled to treat both inorganic and organic components in a unified manner, if so desired, using the same overlapping atoms criterion combined with the appropriate periodic boundary conditions and the minimum image convention.

In this work, we considered MOF systems with well-defined fully occupied positions for all crystallographically determined atoms and used Alvarez’s van der Waals radii to identify bonded and non-bonded adjacent atom pairs. Usually, the coexistence of low-symmetry organic species with the inorganic cage would result in highly complex CEPs, and the corresponding QG(CEP) would be extremely convoluted, while the compression ratios would amount to values such as 50% (in some rare cases, they even exceeded 100%). These results are not without merit, as they convey information in the form of classification of atoms in ‘chemical’ or crystallographic equivalence classes, as previously explained. On the other hand, not all such classes of equivalent atoms are of interest to the chemist who wants to compose known or novel MOFs or to modify existing ones via intervention to specific parts of the lattice.

**Figure 7:**
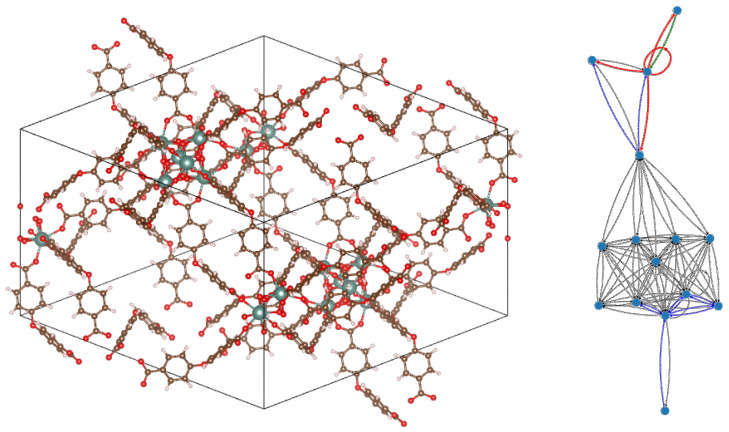
Example of a metal–organic framework (MOF) unit cell with 528 atoms (number of formula units in conventional cell, Z = 6; lattice points per cell, f = 3). Its QG(CEP) contains only 15 nodes and the compression ratio is 2.4%. EPs can squeeze out information (Perron–Frobenius eigenvalue, upper bound of Fiedler eigenvalue, etc.) that would be cumbersome to compute in the original graph. Displayed compound: Catena-[tris(dimethylammonium) tris(μ-4,4${}^{\prime }$-oxydibenzoato)-bis(μ-oxo)-bis(μ-hydroxo)-tri-yttrium(iii) [20]. Left panel: crystal unit cell. Right panel: corresponding QG(CEP), where black = weight 1, blue = weight 2, green = weight 3 and red = weight 4 (original molecular graph omitted, as it would be too complex to allow insightful observations).

One way to simplify the graph representations (both molecular and quotient) would be to disregard the parts of the material that are deemed of lesser interest, e.g., the organic molecules can be replaced by the single lattice points to which they actually correspond (in such a case, though, the adjacency criterion should be carefully redefined). Other alternatives would be to combine CEP with united-atom or functional-group partitioning of the organic species or discarding them altogether and only looking at the inorganic framework. However, we have found that, even without such drastic simplifications, some systems admitted considerable information compression—one such example is shown in Figure 7, where a large MOF unit cell, containing more than 500 atoms, is mapped to a much smaller quotient graph with only 15 vertices, with a compression ratio of a mere 2.4%.

### 3.4. CEP as a Classifier of Materials

The examples in the previous subsections illustrate how CEP classifies atoms of a given molecule or crystal in an insightful manner and provide a quantitative measure of the molecular graph symmetries through the compression ratio of the QG(CEP). The same examples have already demonstrated that CEP is also capable of classifying molecules based on their symmetry. Thus, for instance, all cycloalkanes can be said to form one class, as they share the same CEP. On the other hand, molecular graphs of linear alkanes have a one-to-one correspondence with their CEP, so, in a sense, each linear alkane is a class in its own right; we notice, though, that linear-alkane CEPs in a united-atom representation are also differentiated by the odd or even number of carbon atoms.

We use the above observations to show how CEP and its associated compression ratio can classify materials by clustering them together in different manners, depending on their symmetry. We return to the example of linear and cyclic alkanes in their united-atom representation to illustrate the argument. As previously mentioned, E(EP) is known, from spectral graph theory, to inherit important information from the original graph. The spectrum of the adjacency matrix of QG(EP) is a subset of the one of the molecular graph *G* [10]; the same holds for the Laplacian matrix of QG(EEP) [9]. Furthermore, QG(EP) and *G* share the same Perron–Frobenius eigenvalue, whereas the Fiedler eigenvalue of the former bounds from above the Fiedler eigenvalue of the latter. In the present example, we combine the CEP-based compression ratio with the size of the molecular graph *G* (number of nodes or carbon atoms) and the Perron–Frobenius eigenvalue shared by *G* and its QG(CEP) to investigate the role of equitable partitions and graph spectral information, especially when combined together.

**Figure 8:**
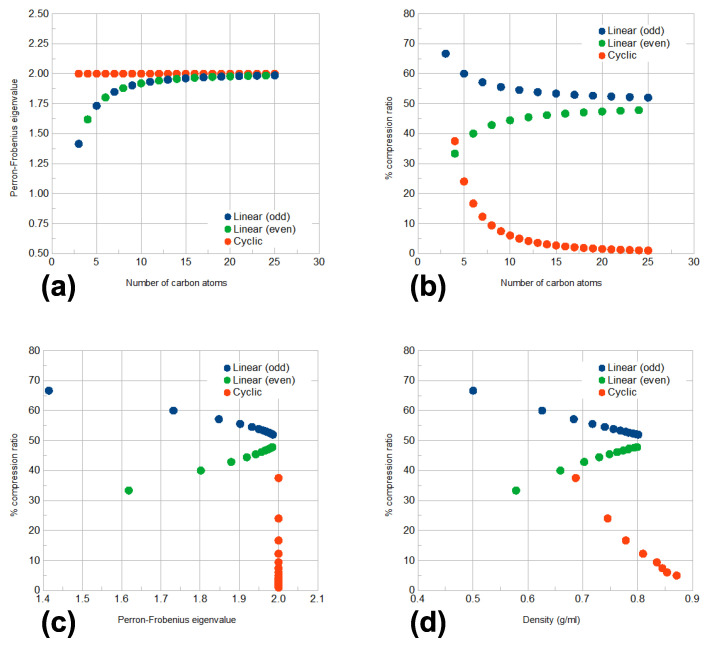
Classification of linear and cyclic saturated hydrocarbons (in their united-atom representation) based on their number of carbon atoms, *n*; Perron–Frobenius eigenvalue, $\lambda_{1}$ shared by their molecular and QG(CEP) graphs; compression ratios, % CR, as defined in Equation (1) and physical properties. Linear hydrocarbons are divided into two groups depending on whether *n* is even (green dots) or odd (blue dots). (**a**) $\lambda_{1}$ vs. *n*; (**b**) % CR vs. *n*; (**c**) % CR vs. $\lambda_{1}$; (**d**) % CR vs. density at ambient conditions.

In Figure 8, the homologous series of linear and cyclic saturated hydrocarbons with three or more carbons are mapped and compared with each other with respect to their number of carbon atoms, *n*; the Perro-n-Frobenius eigenvalue, $\lambda_{1}$, shared by their molecular and quotient graphs and their compression ratio, % CR. We use their united-atom representation (i.e., taking methyl and methylene groups as single, structureless sites) so the molecular graph of a linear hydrocarbon will be a path graph with known spectrum [10,21] given by

(3) $$\lambda_{i}=2\cos\left(\pi i/\left(n+1\right)\right),\;i=1,2,\ldots ,n$$

where $\lambda_{i}$ is the *i*-th eigenvalue. All vertices in these united-atom graphs have the same degree, two, except the two end vertices of the linear molecules, which have degree equal to one. Then, using established bounds for the Perron–Frobenius eigenvalue [21], it is easy to find that $2\left(n-1\right)/n\leq \lambda_{1}\leq 2$ for the path graphs, whereas $\lambda_{1}=2$ for all cycle graphs, regardless of their size.

As Figure 8a–c shows, % CR, combined with the other two descriptors, is able to discern between the two families, linear and cyclic, of hydrocarbon compounds and, even more remarkably, to further divide linear hydrocarbons into two clearly distinct subgroups, according to their symmetry elements that depend on whether *n* is even or odd. Given the known dependence of physical properties of compounds on their size *n*, similar correlations should be preserved when comparing % CR with them. This is demonstrated in Figure 8d, where compression ratios are plotted against hydrocarbon densities at ambient conditions [22,23]. Thus, a quantitative link between molecular symmetries and physical properties can be established with the aid of the compression ratio.

**Figure 9:**
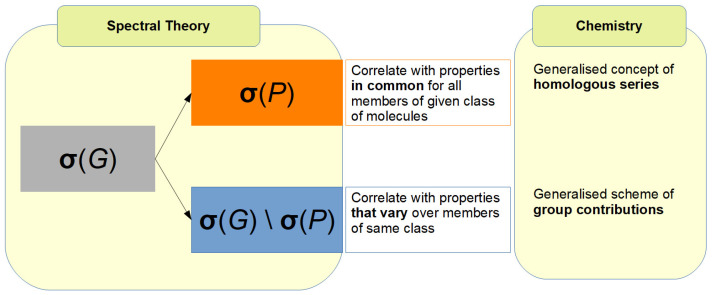
One of the ways to generalise the concepts of *homologous series* and *group contribution* approaches: a correspondence between *spectral graph theory* and *chemistry*. Here, σ(G) denotes the spectrum of a matrix (adjacency, Laplacian, …) associated with graph *G* and σ(P) is the spectrum of the quotient graph arising by applying a partition *P*, such as CEP, to *G*.

Appropriate combinations of CEP-based and spectral information allow chemists to not only classify materials, but also to differentiate the members of each class in an orderly manner, e.g., in the case of alkanes shown in Figure 8. This intra-class ordering of materials based on spectral or other graph-related information can be in direct correspondence with the way their properties vary, in a manner reminiscent of the way the members of a homologous series exhibit varying properties, depending on their order in it. In other words, CEP opens a route to the generalisation of the concept of homologous series. In a similar manner, the well-known approach of *group contribution methods* (as used, for instance, in van Krevelen’s well-known work [24]) can be generalised to a broader framework of *partition-based contributions*. This idea is illustrated in Figure 9 from the perspective of spectral graph theory.

## 4. Discussion

In this work, we have discussed the concept of equitable (and externally equitable) partitions as an aid in the study of the microstructure of molecules and crystalline solid materials. We have defined appropriate adjacency criteria (chemical bonding, nearest neighbours) of atoms in these systems, which allowed us to build graphs that capture the connectivity of the materials at the microscopic level. Then, we introduced the concept of *chemical equitalbe partition* (CEP), which accounts for the chemical composition, as well as connectivity, and was shown to be equitable in the strict sense (akin to a graph divisor). Using an appropriate algorithm, we have been able to derive the CEP and pertinent quotient graphs, QG(CEP), of various molecular and solid crystalline systems. The derivation of these quotient graphs is a kind of information compression operation, which can be described in a quantitative manner; we defined such a measure, herein termed *compression ratio* (% CR). Using simple examples, we demonstrated that CEP classifies the atoms in a system, molecular or other, in manners that make sense to chemists and crystallographers. The compression ratio is a measure of the symmetries inherent in the system’s graph-theoretical representation and can establish a link between these symmetries and the materials’ properties. In particular, when combined with spectral or other graph-related information, it can also classify the materials in many meaningful ways that account for their microstructural symmetries and other features.

Our examples were restricted to systems with well-defined interatomic connectivity. Our future goals include the extensions of the present approach to disordered lattices as well as amorphous systems and molecular liquids and the application to the study of time-varying single- and multi-component model molecular systems generated with the aid of molecular simulation methods. On another level, we are looking to the incorporation of information about geometrical and optical isomerism to molecular graphs. This endeavour may require drastic extensions and changes to the concepts of adjacency and graph equitable partition.

Molecular graph symmetries, which play a key role in our study, should not be confused with molecular or crystal symmetries themselves; the former are related with atom connectivity, whereas the latter concern the arrangement of atoms in space. However, molecular graphs carry, by construction (bonded or nearest neighbours), information about atomic spatial arrangement, albeit in an indirect manner. Thus, graph-related, and molecular and crystal symmetries, are actually facets, partly complementary and partly overlapping, of one and the same ‘reality’ about a system’s microstructure. Thus, establishing links between graph symmetries and physical properties may lead to a novel understanding of structure–property relations in molecular and solid materials. This implies the possibility to back-map properties to graph symmetries and exploit methods of building new graphs possessing similar symmetries [25] as an intermediate step to new molecular structures. If such a scheme works, we opine that our findings—combined with existing experimental, theoretical and modeling methods—may assist in the discovery and design of novel materials with tailor-made properties.

## Figures and Tables

**Table 1:** Construction of the Chemical Equitable Partition for individual molecules through the example of a small linear hydrocarbon (n-propane).

Atom	Step 0	Step 1	Step 2	Step 3	Step 4	Step 5	Step 6
		**1 2**		**1 2 3**		**1 2 3 4**	
C1	1	1 3	1	0 1 3	1	0 1 3 0	1
C2	1	2 2	2	2 0 2	2	2 0 0 2	2
C3	1	1 3	1	0 1 3	1	0 1 3 0	1
H4	2	1 0	3	1 0 0	3	1 0 0 0	3
H5	2	1 0	3	1 0 0	3	1 0 0 0	3
H6	2	1 0	3	1 0 0	3	1 0 0 0	3
H7	2	1 0	3	0 1 0	4	0 1 0 0	4
H8	2	1 0	3	0 1 0	4	0 1 0 0	4
H9	2	1 0	3	1 0 0	3	1 0 0 0	3
H10	2	1 0	3	1 0 0	3	1 0 0 0	3
H11	2	1 0	3	1 0 0	3	1 0 0 0	3

## Data Availability

No new data were created in this study.

## Abbreviations

The following abbreviations are used in this manuscript:

CEP	Chemical Equitable Partition
EEP	Externally Equitable Partition
EP	Equitable Partition
MOF	Metal—Organic Framework
QG	Quotient Graph
QG(*P*)	Quotient Graph of a given partition *P*
